# Arctic foxes as ecosystem engineers: increased soil nutrients lead to increased plant productivity on fox dens

**DOI:** 10.1038/srep24020

**Published:** 2016-04-05

**Authors:** Tazarve Gharajehdaghipour, James D. Roth, Paul M. Fafard, John H. Markham

**Affiliations:** 1 Department of Biological Sciences, University of Manitoba, 50 Sifton Road, Winnipeg, Manitoba R3T 2N2, Canada

## Abstract

Top predators can provide fundamental ecosystem services such as nutrient cycling, and their impact can be even greater in environments with low nutrients and productivity, such as Arctic tundra. We estimated the effects of Arctic fox (*Vulpes lagopus*) denning on soil nutrient dynamics and vegetation production near Churchill, Manitoba in June and August 2014. Soils from fox dens contained higher nutrient levels in June (71% more inorganic nitrogen, 1195% more extractable phosphorous) and in August (242% more inorganic nitrogen, 191% more extractable phosphorous) than adjacent control sites. Inorganic nitrogen levels decreased from June to August on both dens and controls, whereas extractable phosphorous increased. Pup production the previous year, which should enhance nutrient deposition (from urine, feces, and decomposing prey), did not affect soil nutrient concentrations, suggesting the impact of Arctic foxes persists >1 year. Dens supported 2.8 times greater vegetation biomass in August, but δ^15^N values in sea lyme grass (*Leymus mollis*) were unaffected by denning. By concentrating nutrients on dens Arctic foxes enhance nutrient cycling as an ecosystem service and thus engineer Arctic ecosystems on local scales. The enhanced productivity in patches on the landscape could subsequently affect plant diversity and the dispersion of herbivores on the tundra.

Ecosystem engineering can happen at any scale, and due to its ubiquity, at any trophic level^1^. In some cases, physical modification of the environment by ecosystem engineers is relatively large compared to other physical processes operating in the ecosystem (e.g. dam building by beavers). However in most instances, ecosystem engineers are working at a more refined scale. Therefore, separating the effects of an ecosystem engineer from other biotic and abiotic factors is challenging. For example, factors such as species diversity, changes in species distribution, and numerous species interactions within an ecosystem make distinguishing between different biotic influences on soil processes difficult^2^.

Low food web complexity of Arctic biomes, which is primarily due to the bottom-up effect of decreased vegetation diversity and productivity, makes ecosystem engineering easier to study in Arctic communities^3^. Simultaneously, having low species diversity makes Arctic ecosystems highly susceptible to disturbances such as climate warming and human activities^3^. The loss (or introduction) of even a single species can cause drastic and cascading effects in Arctic ecosystems^4^. Therefore, studies on species’ non-trophic impacts (in an ecosystem engineering context) in Arctic biomes are also necessary.

Top predators can drastically change nutrient dynamics of an ecosystem through mechanisms such as decoupling carcass distribution from live-prey distribution. For example brown bears (*Ursus arctos*) feeding on salmon (*Oncorhynchus* spp.) redistribute marine-derived nutrients to terrestrial ecosystems, increasing the forest’s total inorganic nitrogen pools threefold^5^. Carcasses of moose (*Alces alces*) killed by grey wolves (*Canis lupus)* also create hot spots with up to 6 times more inorganic soil nutrients^6^.

The Arctic fox (*Vulpes lagopus*) has a native circumpolar tundra distribution, ranging from northern Greenland (88°N) to the southern edge of Hudson Bay, Canada (53°N). Arctic foxes are top predators, and within their continental range their main prey are microtine rodents including lemmings (*Dicrostonyx* and *Lemmus* spp.) and voles (*Microtus* and *Myodes* spp.)^7^,^8^,^9^. In years with low lemming density, Arctic foxes rely on geese and their eggs during summer^10^, and ringed seal (*Phoca hispida*) pups and carcasses of seals killed by polar bears (*Ursus maritimus*) during winter^11^.

Arctic foxes depend on well-established dens to shelter pups from the harsh Arctic climate and predators^12^. Suitable denning sites for the Arctic fox are mostly on elevated topographical features (e.g. ridges, banks, mounds, moraines) composed of coarse well-draining sediments, and greater depth to permafrost, allowing for easier excavation^13^,^14^. Development of a good den can take many years, with some dens estimated to be hundreds of years old^7^. However, high-quality den sites are limited^15^, and digging new dens is energetically costly and mainly done during peak population years^16^. Climate, soil type, and permafrost also further limit excavations of new dens spatially and temporarily^13^,^12^.

Arctic fox litter size averages 8–10 pups in Canada^12^, so active den sites receive high amounts of nutrients due to urine and faeces deposits as well as nutrient release from the remains of decaying prey items. Due to this nutrient addition, in many Arctic areas, Arctic fox dens have lush green vegetation and are readily spotted across the tundra landscape^13^,^17^,^18^ (Fig. 1). Despite the obvious differences in vegetation growth on Arctic fox dens, studies examining Arctic fox effects on soil are rare. Smith *et al.*^19^ found higher soil total nitrogen (N) levels at den sites compared to off-den areas, but no difference in soil total phosphorous (P). On the Aleutian islands, where the Arctic fox is an introduced species, predation of sea birds by Arctic foxes results in lower guano input, thus fox-inhabited islands have lower soil total N and extractable P (plant available P extracted with Bray extractant) percentages compared to fox-free islands^20^,^21^. A more thorough analysis of the effect of Arctic foxes as chemical ecosystem engineers on soil nutrient dynamics is necessary for better understanding their functional role in nutrient cycling processes in their native range. Specifically, by analysing soil inorganic N and extractable P (as opposed to the total N and P) and seasonal changes in these nutrients, our objective is to estimate the effect of Arctic fox denning activities on local nutrient dynamics.

Primary productivity usually varies more within a tundra site than among sites. This high local variation in primary productivity suggests that soil condition is one of main determinants of primary production in Arctic tundra ecosystems^22^. Primary productivity during short growing seasons in tundra ecosystems is often strongly limited by inorganic N availability in the soil, and followed closely by P, as shown by plant tissue analyses^23^ and fertilization experiments^24^,^25^. Measuring concentrations of the inorganic forms of N and P is necessary for a better understanding of the pool of nutrients available to plants in Arctic tundra where, because of cold temperatures and extremely high or low moisture levels, the decomposition rate of organic material is severely restricted^26^. Although Arctic soils are often rich in organic material and some Arctic plants can make use of the organic form of N, Arctic soils are still fairly poor medium for plant growth due to the fact that organic N in Arctic soil is mostly in insoluble form, and only a small proportion of the soluble organic N occurs in a form that is useable by Arctic plants^27^.

We predicted that, due to nutrient addition by Arctic foxes, inorganic N and extractable P levels at den sites would be higher than control sites, and as a result vegetation biomass would be higher at den sites. Additionally, we predicted that due to receiving nutrients from marine and allochtonous resources in Arctic fox diet (such as geese and seals), δ^15^N values would be elevated in plants growing on fox dens, whereas plants on control sites would have lower δ^15^N signatures, indicative of locally fixed N sources^28^,^29^. We also predicted that dens with pups in the previous year would have higher inorganic N and extractable P levels than dens that did not have pups.

## Results

**Figure 2:**
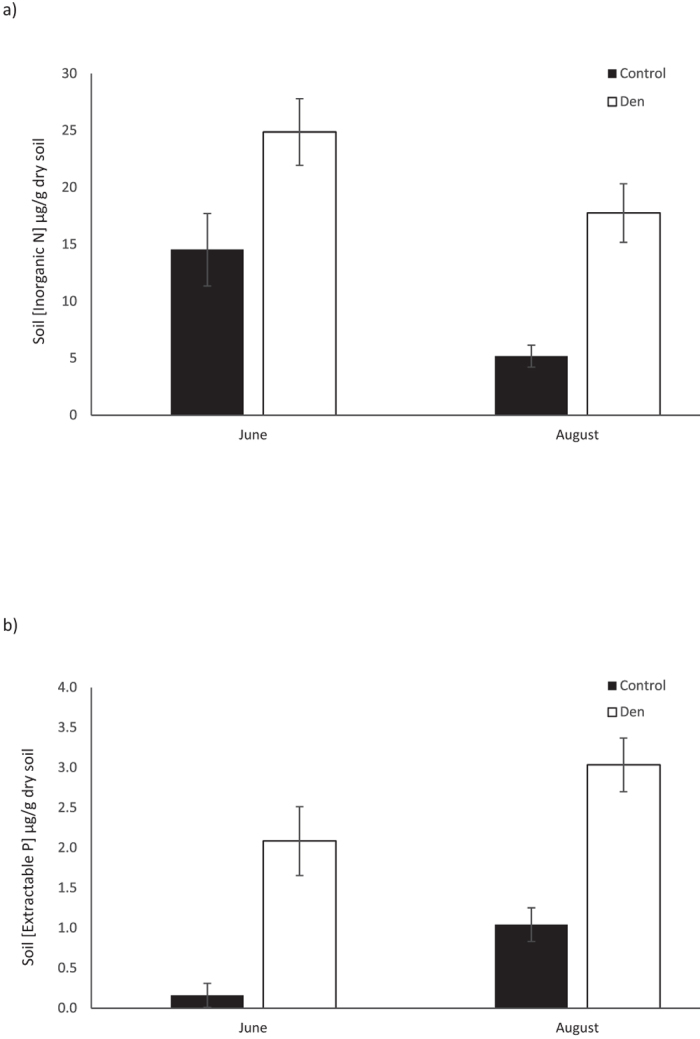
Nutrient concentrations (mean ± s.e.m.) of soil samples collected from fox dens and control sites in Wapusk National Park, Canada, in June and August 2014. (**a**) Inorganic N (n = 17) (**b**) Extractable P (n = 11).

Den sites had 71% more soil inorganic N than control sites in June. In August, the difference in inorganic N concentration between den and control sites increased threefold, to 242% (Fig. 2a, Supplementary Table S1). The mixed effect analysis on inorganic N data indicated that site and season both had a significant effect (χ^2^_site (1)_ = 27.763, p_site_ < 0.0001; χ^2^_season (1)_ = 13.48, p_season_ = 0.0002). Inorganic N concentration in soil was higher on dens compared to control sites (β = 1.675), and higher in June compared to August (β = 1.087). In the full model, marginal R^2^ = 0.407 and conditional R^2^ = 0.476. In June, the nitrate to ammonium ratio was higher on control sites (2.677 ± 0.744, mean ± s.e.m.) than den sites (1.792 ± 0.811) (t_(16)_ = 2.470, p = 0.0126). Nitrate to ammonium ratio did not differ between dens (1.162 ± 0.158) and control sites (1.320 ± 0.254) (t_(16)_ = 0.439, p = 0.333) in August.

Extractable P levels in soils were 1195% higher on dens compared to control sites in June, and 191% higher in August. However, unlike inorganic N levels, extractable P levels were higher in August than in June (Fig. 2b, Supplementary Table S1). Mixed effect analysis results suggest that extractable P concentration also differed between sites and seasons (χ^2^_site (1)_ = 32.89, p_site_ < 0.0001; χ^2^_season (1)_ = 19.15, p_season_ < 0.0001). Extractable P concentration was greater on dens than control sites (β = 0.826) and lower in June compared to August (β = −0.428). Marginal R^2^ and conditional R^2^ values were 0.657 and 0.770 respectively for the full model.

The presence of fox pups the previous summer did not affect soil inorganic N (χ^2^_(1)_ = 0.092, p = 0.76) or P concentrations (χ^2^_(1)_ = 0.306, p = 0.58).

δ^15^N values of *L. mollis* did not differ between dens (2.78 ± 0.94 per mil, mean ± s.e.m.) and their paired control sites (3.56 ± 0.958 per mil) (t_(14)_ = −0.555, p = 0.37). Soil coarse fraction also did not differ between dens (0.077 ± 0.008) and paired control sites (0.091 ± 0.014) (χ^2^_(1)_ = 0.003, p = 0.956).

**Figure 3:**
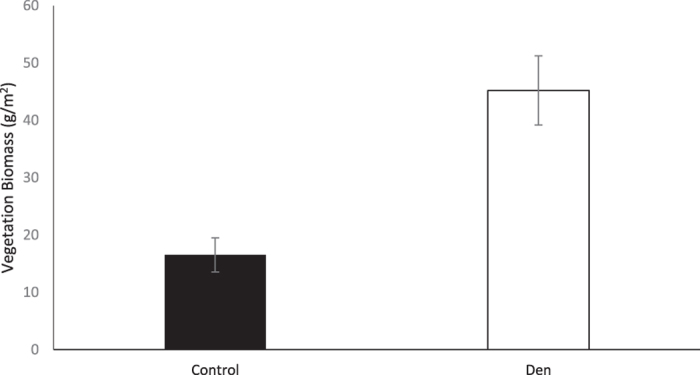
Vegetation biomass on fox dens and control sites in Wapusk National Park, Canada, in August, 2014 (mean ± s.e.m., n = 18).

Den sites (191.251 ± 23.667 g) were more productive than control sites (68.907 ± 10.925 g) (t_(17)_ = 4.531, p = 0.0002). Dens on average supported 2.8 times more vegetation biomass than controls (Fig. 3).

## Discussion

Enriched inorganic N and extractable P levels in soil and increased plant biomass at den sites compared to control sites indicate that Arctic fox denning activity provides vegetation with the limiting factor for their growth. Average total inorganic N concentrations on control sites in both June and August were comparable to background concentrations reported for similar Dryas heath tundra ecosystems^30^ (Fig. 2a). Higher inorganic N levels in June compared to August suggest that the growing vegetation is using the available inorganic N in the soil. Furthermore, in similar Arctic ecosystems, inorganic N peaks early in spring immediately or slightly after snowmelt, and then progressively declines throughout the summer^31^. Due to nutrient deposition by foxes, dens maintain a more stable nitrate to ammonium ratio throughout the growing season compared to control sites. Control sites experience a 2 fold decrease in nitrate to ammonium ratio caused by a 3.5 fold drop in nitrate levels due to leaching (Supplementary Table S1).

Even after uptake of N by plants in August, den areas still have higher inorganic N content than control sites. Furthermore, in August the difference in inorganic N concentrations between den and control sites is greater than in June (Fig. 2a). These findings suggest that the nutrient enriching effects of Arctic foxes as ecosystem engineers is strong enough to support vegetation biomass 2.8 times as high as control sites (Fig. 3), and simultaneously elevate unused inorganic N concentrations on dens far above concentrations on controls.

Our estimates of the difference in annual plant productivity between den and control areas are conservative. Unlike *L. mollis* and willow species, *D. integrifolia* is an evergreen species^32^, thus not all the above-ground *D. integrifolia* biomass collected is produced in one growing season. Furthermore, *L. mollis* dominates on the den sites but it is almost non-existent on control sites, whereas *D. integrifolia* dominates on the control areas and is not commonly found in high proportions on den sites (Fafard unpubl. data). Therefore, the effect of Arctic fox nutrient deposition on plant biomass is most likely even greater than our estimates.

Average extractable P concentrations on control sites were similar to concentrations suggested in previous studies^31^ (Fig. 2b). A number of studies on P dynamics in Arctic ecosystems have concluded that one of the largest pools of potentially available P in Arctic soils is the microbial biomass, which immobilizes P during the growing season^33^. Microbial biomass can contain up to about 35% of total P pool, compared to only ~3.5% of total N pool. Therefore, lower levels of microbial immobilization of P is likely the reason for higher concentration of extractable P in August than in June. Additionally, increased depth to permafrost and soil temperatures on Arctic fox dens compared to control sites^19^ could further enhance the microbial activities involved in nutrient mineralization processes^26^.

Soil coarse fraction can affect drainage within the soil, which can in turn influence soil nutrient content and plant growth^34^. Smith *et al.*^19^ found that Arctic fox dens located on the Yukon Coastal Plain and Herschel Island were sandier than off-den sites. However in our study area, soil coarse fraction did not differ between dens and paired control sites. Thus differences in soil nutrient and vegetation biomass content between den and control sites are not due to different soil textures

By constantly depositing nutrients, Arctic foxes maintain the higher inorganic N and extractable P concentrations on den sites compared to control sites. This effect could be carried on for multiple years, considering that dens with and without pups did not differ in inorganic N and extractable P concentrations. Furthermore, the enhancing effect of foxes on nutrient levels does not seem to be restricted to the growing season, since we found fox urine on about 80% of these dens in April (Roth, unpubl. data), suggesting that dens are visited regularly throughout the year.

Nitrogen to phosphorus (N:P) ratios in plant tissues are regularly used as reliable indicators of nutrient limitation for both vascular plants^35^ and bryophytes^36^. N:P ratios greater than 16 suggest P limitation, whereas N:P ratios less than 14 indicate N limitation and N:P ratios between 14 and 16 indicate N and P co-limitation^35^,^37^. However, due to homeostatic regulation by plants, N:P ratios in plants are not equal to N:P ratios in soil. Homeostatic regulation coefficients (the inverse slope of the log-log relationship between soil and plant N:P) can vary from 1.7 to 4.6^38^, and based on our measured soil N:P ratios, control sites are likely P limited in June, and N limited in August. Den sites, however, are likely to be N limited in both seasons.

δ^15^N values in grass samples from den and control areas were not different, contrary to our predictions. One possible explanation is the discrimination against the heavier N isotope during N mineralization process (and other soil N transformations)^39^. Studies have also found that if the N pool is large enough, mineralization and uptake rate do not affect its size, and the discrimination against ^15^N plant uptake processes is more pronounced^40^. Inorganic N levels only decreased by 26% from June to August on den soils, and compared to control sites, inorganic N levels are maintained at a high level throughout the growing season on dens. As a result, ^15^N discrimination during plant uptake could be more pronounced on dens than control sites, where even after the minimal vegetation growth, inorganic N decreases by 62%. As heavier N isotopes from consumed prey usually get incorporated in the predator’s body, with lighter isotopes flushed out in urine^41^, our results could also suggest that deposition of urine by Arctic foxes is potentially a more important source of nutrients for vegetation growth than decomposing prey items such as geese (with high δ^15^N values) in Arctic areas, where decomposition rates are slow.

In conclusion, our study shows that Arctic foxes engineer Arctic ecosystems on local scales: through nutrient deposition, Arctic foxes change the soil N and P dynamics. Increased inorganic N and P concentrations on Arctic fox dens improved conditions on the tundra for plant growth, sustaining high vegetation biomass. Nutrient cycling is considered to be one of the most important ecosystem services^42^, and our results suggest that by generating spatial heterogeneity in nutrient distributions, Arctic foxes, as top predators, exert strong positive influences on ecosystem nutrient dynamics. These positive influences feed upward to increase vegetation productivity and landscape heterogeneity. By enhancing nutrient dynamics locally, Arctic foxes could have an important role in providing ecosystem services in Arctic tundra landscape^43^. Range contraction of Arctic foxes due to climate warming and the encroachment of red foxes (*Vulpes vulpes*) could result in the loss of the ecosystem services they provide.

Besides increasing vegetation biomass, the nutrient enhancement on fox dens could also affect plant biodiversity and potentially may attract herbivores to Arctic fox den sites. In fact lemming droppings and reindeer droppings have been observed at Arctic fox dens sites in Alpine tundra^18^. As mammalian herbivores in Arctic tundra can elevate soil nitrogen and phosphorous concentrations through deposition of their waste products^44^, their use of Arctic fox dens could further enhance local nutrient dynamics. Additional exploration of the influence of Arctic foxes on herbivore communities through mechanisms other than predation would broaden the scope of understanding the role of Arctic foxes as ecosystem engineers.

## Methods

Our study was conducted in the western Hudson Bay coastal habitat within Wapusk National Park, Manitoba, Canada. This region is close to the southern boundary of Arctic fox distribution in North America^45^. Retraction of the ice load, followed by post-glacial rebound of the land, has made sand or gravel north-south oriented beach ridges one of the main landform features in this region^46^,^47^. These elevated beach ridges are separated by many depressed shallow lakes and ponds^46^. Greater depth to permafrost compared to the surrounding lowlands, and xeric moisture levels due the sandy texture of the soil, make these beach ridges suitable Arctic fox denning habitat^7^,^13^,^48^,^49^. Vegetation on beach ridges is typical of low-growing heath communities^50^; specifically *Dryas* heath due to the major dominance of *Dryas integrifolia*^51^. However, Arctic fox dens are usually covered with lush green vegetation, dominated by *Leymus mollis*, a perennial deciduous graminoid that can colonize subarctic coastal dunes^52^, and *Salix planifolia*, a perennial deciduous shrub and native colonizer of primary succession tundra^53^. Since 2010 we have visited all know dens in the area in April, June, and August each year to examine them for signs of fox activity. Fresh prey items, tracks, faeces, signs of digging and numerous cleared out burrows reflect the presence of pups in August^54^.

Soil samples were collected from fox dens in June and again in August, 2014. We collected 5 samples from each den: 1 sample was collected from the centre of the den (the midpoint of a straight line connecting the two farthest open burrows) and 4 samples were collected 5 meters from the centre, in opposite directions (2 parallel to the beach ridge and 2 perpendicular to the beach ridge). For each den site a paired control area was chosen at a similar elevation, slope and aspect. The centre of the control area was designated 50 m away from the centre of the den to ensure that control area was well outside of the fox denning area. For each control site, 5 samples were collected following the same protocol as den samples. Each sample was a 10 cm deep cone of soil (approximately 200 cm^3^ in volume) kept frozen until analysis.

To estimate productivity we collected vegetation biomass samples in August. Although not all plant species in our area are deciduous, variation in vegetation biomass should reflect variation in productivity. At each sampling location, a 1 m^2^ quadrat was placed centred on the soil sampling hole, and the above ground live plant biomass was collected from the northeast quarter of the quadrat. For *D. integrifolia*, however, we collected this species from only 13 sampling locations, estimated its percent cover from those locations, and used the relationship between biomass and percent cover (biomass = −1.730 ± 0.597* percent cover, F_1,11_ = 86.418, p < 0.0001, R^2^ = 0.887) to estimate biomass at the remaining sampling locations based on percent cover. When possible, *L. mollis* samples were also collected from den and paired control sites for stable isotope analysis.

Each soil sample was thawed in the lab and homogenized, and a subsample was air dried. Total inorganic N concentrations [NH_4_^++^NO_3_^−^] were measured using the accelerated microdiffusion method^55^ and extractable P concentrations [PO4^−^] were determined from sodium bicarbonate extracts using the Murphy Riley technique^56^. Soil coarse fraction of August samples, reflecting soil texture, was measured by separating particles larger than 2 mm (gravel) and particles 2mm in size or smaller (sand). To estimate productivity, vegetation biomass samples were dried to constant weight. *L. mollis* samples were also dried to constant weight and homogenized with a ball mill. δ^15^N values were measured in 3 mg subsamples using an elemental analyzer and a continuous flow isotope ratio mass spectrometer at the University of Windsor.

To compare soil inorganic N and P concentrations between 17 and 11 den and control pairs respectively, and between seasons (June vs. August), we performed linear mixed effect analysis in R^57^, using lme4 package^58^. Site (den vs. control) and season (without the interaction term) were entered as fixed effects into the model. As a random effect, we let intercepts vary for site id. To satisfy the normality and homoscedasticity assumptions, inorganic N and P concentrations for the 5 samples at each site were averaged and square-root transformed. To obtain p-values for each fixed effect, likelihood ratio tests of the full model against reduced models without the effect in question were used. Marginal R^2^ (proportion of variance explained by the fixed factors) and conditional R^2^ (proportion of variance explained by both the fixed and random factors) values were calculated based on Nakagawa & Schielzeth^59^ using lme4^58^ and arm^60^ packages. Nitrate to ammonium ratio ([NO_3_^−^]/[NH_4_^+^]) was averaged for each site, and compared separately for each season between 17 den and control pairs using paired t-test. Soil coarse fraction was compared between 20 den and control sites using a Kruskal-Wallis test.

Vegetation biomass measurements were averaged for each site and then compared between 18 den and control pairs using paired t-test. To determine if dens receive N from allochtonous sources, δ^15^N values were averaged for each site, and then compared between 15 den and control pairs, using paired t-test. To investigate the effect of pup presence on soil inorganic N (n = 34) and P (n = 22) concentrations a linear mixed effect model was constructed; season and pup presence were entered as fixed factors and site id as a random factor. Inorganic N and P values were square root transformed to satisfy the normality and homoscedasticity assumptions. The full model was then compared to the reduced model (i.e. model without pup presence as a factor) using a likelihood ratio tests.

## Additional Information

**How to cite this article**: Gharajehdaghipour, T. *et al.* Arctic foxes as ecosystem engineers: increased soil nutrients lead to increased plant productivity on fox dens. *Sci. Rep.* **6**, 24020; doi: 10.1038/srep24020 (2016).

## Supplementary Material

Supplementary Information

## Figures and Tables

**Figure 1:**
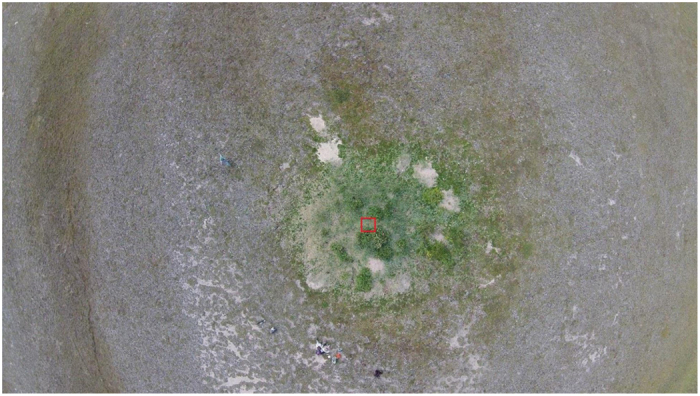
Aerial photo of an Arctic fox den in Wapusk National Park, Canada, in August 2014, showing the contrast between the lush green vegetation on dens (dominated by *Leymus mollis* and *Salix planifolia*) and the background *Dryas* heath on beach ridges. For scale, a 1 × 1 m quadrat can be seen in the middle of the den.
