# Supplementary material for: Arctic foxes as ecosystem engineers: increased soil nutrients lead to increased plant productivity on fox dens
Source: Sci Rep. 2016 Apr 5;6:24020. doi: 10.1038/srep24020 (PMC4820751; doi:10.1038/srep24020)
Supplement: Supplementary Information [file srep24020-s1.pdf]

Arctic foxes as ecosystem engineers: increased soil nutrients lead to increased plant productivity on fox dens

Tazarve Gharajehdaghipour, James D. Roth, Paul M. Fafard & John H. Markham

Supplementary Information

Table S1: Nutrient concentrations (µg/g dry soil, mean ± s.e.m.) of soil samples collected from fox dens and control sites in Wapusk National Park, Canada, in June and August 2014.

	Control-June	Den-June	Control-August	Den-August
Ammonium	4.269 ± 0.768	12.455 ± 1.932	2.225 ± 0.388	9.126 ± 1.865
Nitrate	10.271 ± 2.940	12.417 ± 2.615	0.646 ± 1.293	8.631 ± 1.293
Total inorganic nitrogen	14.540 ± 3.185	24.871 ± 2.926	5.191 ± 0.967	17.758 ± 2.566
Extractable phosphorous	0.161 ± 0.150	2.086 ± 0.429	1.044 ± 0.210	3.036 ± 0.334
